# High Spatial Resolution and Temporally Resolved T_2_^*^ Mapping of Normal Human Myocardium at 7.0 Tesla: An Ultrahigh Field Magnetic Resonance Feasibility Study

**DOI:** 10.1371/journal.pone.0052324

**Published:** 2012-12-14

**Authors:** Fabian Hezel, Christof Thalhammer, Sonia Waiczies, Jeanette Schulz-Menger, Thoralf Niendorf

**Affiliations:** 1 Berlin Ultrahigh Field Facility (B.U.F.F.), Max Delbrueck Center for Molecular Medicine, Berlin, Germany; 2 Experimental and Clinical Research Center, a Joint Cooperation between the Charité Medical Faculty and the Max Delbrueck Center for Molecular Medicine, Campus Berlin Buch, Berlin, Germany; 3 Department of Cardiology and Nephrology, HELIOS Klinikum Berlin Buch, Berlin, Germany; University Hospital of Würzburg, Germany

## Abstract

Myocardial tissue characterization using T_2_^*^ relaxation mapping techniques is an emerging application of (pre)clinical cardiovascular magnetic resonance imaging. The increase in microscopic susceptibility at higher magnetic field strengths renders myocardial T_2_^*^ mapping at ultrahigh magnetic fields conceptually appealing. This work demonstrates the feasibility of myocardial T_2_^*^ imaging at 7.0 T and examines the applicability of temporally-resolved and high spatial resolution myocardial T_2_^*^ mapping. In phantom experiments single cardiac phase and dynamic (CINE) gradient echo imaging techniques provided similar T_2_^*^ maps. *In vivo* studies showed that the peak-to-peak B_0_ difference following volume selective shimming was reduced to approximately 80 Hz for the four chamber view and mid-ventricular short axis view of the heart and to 65 Hz for the left ventricle. No severe susceptibility artifacts were detected in the septum and in the lateral wall for T_2_^*^ weighting ranging from TE = 2.04 ms to TE = 10.2 ms. For TE >7 ms, a susceptibility weighting induced signal void was observed within the anterior and inferior myocardial segments. The longest T_2_^*^ values were found for anterior (T_2_^*^ = 14.0 ms), anteroseptal (T_2_^*^ = 17.2 ms) and inferoseptal (T_2_^*^ = 16.5 ms) myocardial segments. Shorter T_2_^*^ values were observed for inferior (T_2_^*^ = 10.6 ms) and inferolateral (T_2_^*^ = 11.4 ms) segments. A significant difference (p = 0.002) in T_2_^*^ values was observed between end-diastole and end-systole with T_2_^*^ changes of up to approximately 27% over the cardiac cycle which were pronounced in the septum. To conclude, these results underscore the challenges of myocardial T_2_^*^ mapping at 7.0 T but demonstrate that these issues can be offset by using tailored shimming techniques and dedicated acquisition schemes.

## Introduction

Emerging cardiovascular magnetic resonance (CMR) imaging applications include T_2_^*^ relaxation sensitized techniques, which are increasingly used in basic research and (pre)clinical imaging. Methodological developments in T_2_^*^ sensitized imaging [1]–[4] and simulations of myocardial vasculature [5], [6] have been indispensable. Applications include investigation of the microstructure of the isolated rat heart [7], detection of myocardial ischemia [8]–[14], probing of vasodilator or dipyridamole-induced changes in myocardial perfusion [15]–[18], visualization of scarred myocardium [19], imaging of capillary recruitment [20] and assessment of tissue oxygenation related to endothelium-dependent blood flow changes [21]. T_2_^*^ mapping has also been shown to be of substantial clinical value for the ascertainment of myocardial iron levels [22]–[29].

The most widely used methods for T_2_^*^ mapping are echo planar imaging (EPI) and gradient echo based techniques. Unlike conventional CINE gradient echo imaging, the relatively strong T_2_^*^-weighting required to make gradient echo sequences sensitive to changes in magnetic susceptibility asks for a long evolution time (TE) between RF excitation and data acquisition. Consequently, gradient echo based myocardial T_2_^*^ mapping is commonly restricted to a single slice and single cardiac phase that can be accommodated in a single breath-hold at 1.5 T and 3.0 T [30]–[32].

The linear relationship between magnetic field strength and microscopic susceptibility [33]–[35] renders it conceptually appealing to pursue myocardial T_2_^*^ mapping at ultrahigh magnetic field strengths. Realizing the opportunities and challenges of T_2_^*^ mapping, this pilot study demonstrates the feasibility of ultrahigh field susceptibility-weighted myocardial imaging and examines its applicability for temporally-resolved and high spatial resolution myocardial T_2_^*^ mapping at 7.0 T. To meet this goal, the applicability of 2D spoiled gradient-echo multi-echo based techniques for T_2_^*^ mapping at 7.0 T is closely investigated in phantom experiments. The feasibility of gradient-echo multi-echo based techniques for fast CINE T_2_^*^ mapping of the human heart is demonstrated at 7.0 T. We also present the suitability of this technique for high spatial resolution myocardial T_2_^*^ mapping by using thin slices (slice thickness = 2.5 mm) and in-plane spatial resolution of (1.1×1.1) mm^2^. Our initial volunteer studies serve as a mandatory precursor to a broader clinical study. The merits and limitations of T_2_^*^ mapping using 2D spoiled gradient-echo multi-echo imaging at 7.0 T are discussed and implications for cardiac MR at 7.0 T are considered.

## Methods

### MR-Hardware

Imaging was conducted using a 7.0 T whole body MR scanner (Magnetom, Siemens Healthcare, Erlangen, Germany) equipped with a gradient system (Avanto, Siemens Healthcare, Erlangen, Germany) capable of supporting a slew rate of 200 mT/m/ms and a maximum gradient strength of 40 mT/m. A 16 channel transmit/receive coil array was used for excitation and signal reception. The coil was designed for cardiac imaging and comprises an anterior and posterior former, each laid out on a two-dimensional 2 by 4 grid of loop elements. For further details about the coil please see [36], [37]. An MR stethoscope (EasyACT, MRI.TOOLS GmbH, Berlin, Germany) was used for cardiac triggering [38], [39].

### T_2_^*^ Mapping Techniques

**Figure 1:**
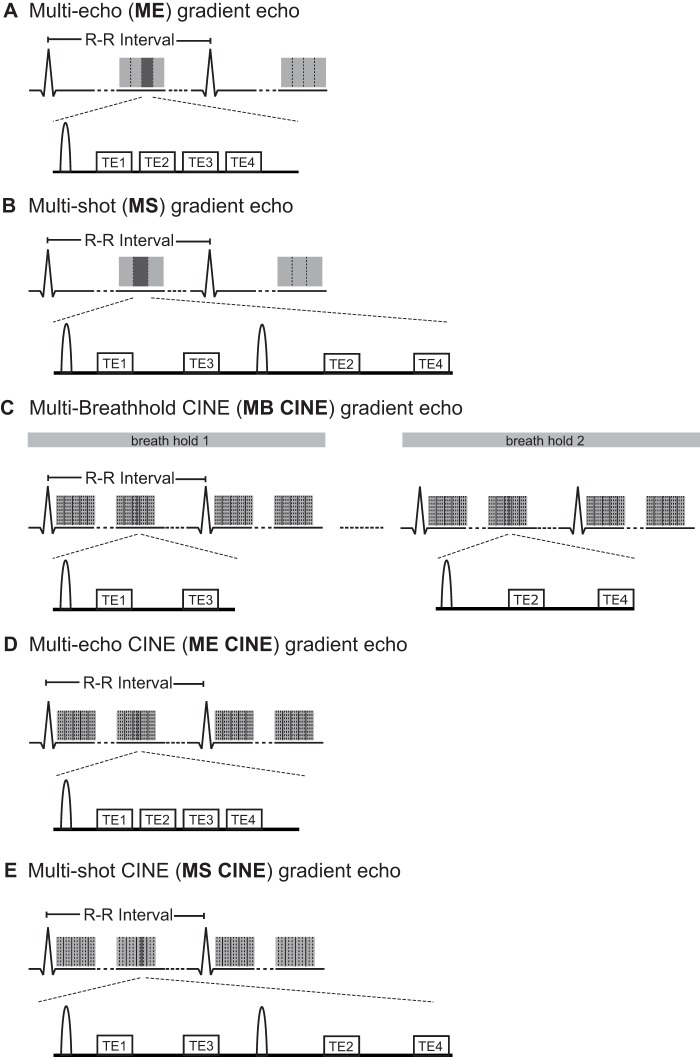
Synopsis of multi-echo gradient echo strategies used for T_2_^*^ mapping at 7.0 T. **A).** Conventional multi-echo (**ME**) gradient echo for single cardiac phase myocardial T_2_^*^ mapping. Multiple echoes are acquired after excitation to obtain a set of T_2_^*^ weighted images. The competing constraints of inter echo time and spatial resolution inherent to the **ME** approach are addressed by the **B**) interleaved multi-shot multi-echo (**MS**) gradient echo technique. In **MS** a set of excitations is employed together with echo interleaving echoes to acquire a set of T_2_^*^ weighted images. **C**) The multi-breath-hold multi-echo (**MB CINE**) gradient echo technique allows myocardial CINE T_2_^*^ mapping by interleaving the echoes over several breath-holds. For benchmarking **D**) multi-echo CINE (**ME CINE**) gradient echo and **E**) multi-shot multi-echo CINE (**MS CINE**) were applied for T_2_^*^ mapping in phantom studies. To guide the eye vertical dashed lines refer to k-space lines. Vertical solid lines refer to cardiac phases. A unipolar readout using gradient flyback was applied for all strategies.

Myocardial T_2_^*^ mapping is commonly conducted with cardiac triggered, segmented multi-echo spoiled gradient echo (**ME**) techniques that use breath-held acquisitions for respiratory motion compensation [8], [9], [15], [18], [40]. In this work, various ME configurations have been used for (i) time resolved CINE and for (ii) single cardiac phase acquisitions (Figure 1).

For single cardiac phase imaging, the acquisition period is commonly placed into end-diastole, which limits the viable window of data acquisition to 100 ms to 200 ms. Data acquisition is segmented over a series of cardiac cycles with each segment acquiring a set of echoes during the quiescent interval (Figure 1a). The number of segments and echoes per segment are dictated by the longest TE used for T_2_^*^ weighting as outlined in Figure 1a. To avoid T_2_^*^ errors due to signal modulations induced by fat-water phase shift, it is essential to choose echo times where fat and water are in-phase [4]. At 1.5 T and 3.0 T TE increments equivalent to the fat-water shift are 4.4 ms and 2.2 ms, respectively. At 7.0 T this inter echo time is 1.02 ms, which is beneficial for rapid multi-echo acquisitions. However, if a larger data matrix size is needed for high spatial resolution T_2_^*^ mapping, the readout/acquisition window can easily exceed 1.02 ms even when short dwell times are used. Consequently, it is elusive to accomplish inter-echo time increments of 1.02 ms for high spatial resolution T_2_^*^ mapping at 7.0 T using sequential multi echo gradient echo imaging.

CINE T_2_^*^ mapping might be feasible at 7.0 T assuming a T_2_^*^ reduction at 7.0 T versus 1.5 T and 3.0 T and considering the relationship between proper T_2_^*^ weighting and range of echo times to be covered. To this end, a maximum TE = 10 ms would be compatible with the needs of CINE imaging but would also provide sufficient coverage of the T_2_^*^ decay at 7.0 T.

For all these reasons, two imaging strategies were employed at 7.0 T:

- Interleaved multi-shot multi-echo (**MS**) gradient echo technique for single cardiac phase myocardial T_2_^*^ mapping (Figure 1b). This approach addresses the competing constraints of inter echo time and spatial resolution of the **ME** approach by adding more excitations and by interleaving the echoes.
- Multi-breath-hold multi-echo (**MB CINE**) gradient echo technique for CINE myocardial T_2_^*^ mapping (Figure 1c). This approach runs the trait that all k-space lines required to form the final image for a given echo time are acquired in a single breathhold.

### Phantom Studies

For the evaluation of **MS** and **MB CINE** T_2_^*^ mapping strategies, phantom experiments were conducted using a long T_2_^*^ and medium T_2_^*^ phantom. For long T_2_^*^, a cylindrical water phantom (diameter = 15 cm) containing an agarose copper sulfate solution (4 g CuS0_4_ +2 g NaCl +2 g agarose dissolved in 1.0 l H_2_O) was used. A glass capillary (inner diameter  = 0.5 mm) filled with air and a tube (inner diameter  = 5 mm) filled with water were placed inside the phantom to create strong susceptibility gradients of limited spatial extension within the uniform phantom. For the medium T_2_^*^ phantom, a cylindrical water phantom (diameter = 8 cm) containing agarose (5 mg agarose dissolved in 250 ml H_2_O) was used. T_2_^*^ was reduced by ultrasmall superparamagnetic iron oxide particles (500 µl Molday ION (10 mg Fe/ml), BioPal, Worchester, USA), which afforded a T_2_^*^ of approximately 20 ms.

In the phantom experiments, **MS** (Figure 1b) and **MB CINE** (Figure 1c) T_2_^*^ mapping strategies were benchmarked against other T_2_^*^ mapping techniques, which are already established at 1.5 T and 3.0 T but are unsuitable for myocardial T_2_^*^ mapping at 7.0 T due to echo time and acquisition time constraints. These reference methods include:

- conventional multi-echo (**ME**) gradient echo for single cardiac phase T_2_^*^ mapping (Figure 1a).
- multi-echo CINE (**ME CINE**) gradient echo (Figure 1d).
- multi-shot multi-echo CINE (**MS CINE**) gradient echo (Figure 1e).

For phantom T_2_^*^ mapping, an image matrix of 320×240, a field of view of (360×270) mm^2^, an in-plane resolution of (1.1×1.1) mm^2^, and a slice thickness ranging from 2.5 mm to 8 mm were used. A unipolar readout using gradient flyback was applied together with echo times ranging from 2.04 ms to 10.20 ms. This approach results in nine equidistant echoes with an inter echo time of 1.02 ms with the exception of **ME** and **ME CINE** due to gradient switching induced peripheral nerve stimulation constraints. For **ME** and **ME CINE** 6 echoes with an inter echo time of 3.06 ms and a TE_min_ = 2.04 ms were used. For the **MS**, **MS CINE** and **MB CINE** techniques three excitations together with 3 echoes were used to ensure an inter-echo time of 1.02 ms. With the first excitation echo 1, 4 and 7 were acquired. The second excitation covered echo 2, 5 and 8 while echo 3, 6, and 9 were recorded after the third excitation. A simulated heart rate of 60 bpm was used for prospective triggering of the phantom experiments.

### Ethics Statement

For the *in vivo* feasibility study, 8 healthy subjects (mean age: 27±3 years, 5 females, mean BMI: 24 kg/m^2^, mean heart rate: 78 bpm) without any known history of cardiac disease were included after due approval by the local ethical committee (registration number DE/CA73/5550/09, Landesamt für Arbeitsschutz, Gesundheitsschutz und technische Sicherheit, Berlin, Germany). Informed written consent was obtained from each volunteer prior to the study.

### Volunteer Studies

For each volunteer, slice positioning was carried out following international consensus by the same technician to omit inter-operator variability. Myocardial T_2_^*^ mapping was conducted using the **MS** and the **MB CINE** imaging strategies for all subjects. For this purpose, a mid-ventricular short axis view and a four chamber view were used. Imaging parameters were set to: acquisition data matrix  = 256×224, FOV  =  (288×252) mm^2^, in-plane resolution  =  (1.1×1.1) mm^2^, slice thickness = 4 mm if not otherwise stated. A nominal flip angle of α = 20° has been applied; and electro magnetic field (EMF) simulations using the human voxel model “Duke” were conducted for transmission field shaping to enhance B_1_^+^ uniformity across the heart [36], [37], [41]. Echo times ranging from 2.04 ms to 10.20 ms were applied. Three excitations together with 3 echoes per excitation were used to ensure an inter-echo time of 1.02 ms. With the first excitation, echo 1, 4 and 7, were acquired. The second excitation covered echo 2, 5 and 8. Echo 3, 6, and 9 were recorded after the third excitation. Moderate acceleration (R = 2 for **MS** and R = 3 for **MB** **CINE**) in conjunction with GRAPPA reconstruction [42] was applied to reduce the breath-hold time. For single cardiac phase acquisitions, the **MS** protocol was prospectively triggered to place data acquisition at end-diastole or end-systole. For prospectively triggered CINE T_2_^*^ mapping, 25 cardiac phases were acquired for a heart rate of 60 bpm. Phase images of the first two echoes (TE_1_ = 2.04 ms, TE_2_ = 3.06 ms) of **MS** were used to determine B_0_ field maps offline.

Prior to T_2_^*^ mapping, volume selective B_0_ shimming was conducted to reduce static magnetic field inhomogeneities [43], [44]. In doing so, the susceptibility weighting will be dictated by microscopic B_0_ susceptibility gradients, rather than by macroscopic B_0_ field inhomogeneities. For this purpose, a 2D multi-slice, cardiac gated, breath-hold double echo (**DE**) gradient echo sequence (TE_1_ = 3.06 ms, TE_2_ = 5.10 ms) was used for B_0_ field mapping. Cardiac gating and breath-holding were applied to reduce and possibly eliminate phase contributions induced by cardiac and respiratory motion. The shim volume was adjusted to cover the left and right ventricle in the four chamber view and in the short axis view of the heart. Data acquisition for B_0_ shimming was adjusted to diastole. This choice is based on previous reports which demonstrated that field maps showed a negligible temporal variation across the cardiac cycle [43]. For the shim volumes, linear and second order room temperature shims were calculated to reduce the frequency shift across the phantom or across the heart with the goal to render B_0_ uniform. The overall protocol time, including localizer, slice angulation, volume selective B_0_ mapping routine, 2D FLASH CINE imaging ( 4 chamber view and short axis view) and T_2_^*^ mapping using MS and MB CINE was approximately 30 minutes. Table 1 outlines the breath hold times used for MS and MB CINE T_2_^*^ mapping in healthy volunteers.

**Table 1:** Synopsis of scan time duration and temporal resolution used for the single cardiac phase and CINE T_2_^*^ mapping protocols.

	ME	MS	MB CINE	ME CINE	MS CINE
**scan duration phantom study**	25s	49s	3×81s	121s	241s
**scan duration ** ***in vivo*** ** study**	-	24s	3×22s	-	-
**acquisition window length**	205ms	192ms	36ms	38ms	36ms

The scan duration of **MS** protocols is doubled versus **ME** protocols. For *in vivo* T_2_^*^ mapping **MS** and **MB** **CINE** were applied. CINE protocols provided an acquisition window length of about 36 ms, which renders the impact of cardiac motion effects rather low. The acquisition window length is given by the number of k-spaces lines acquired per cardiac cycle times the repetition time.

### Post-Processing and Image Analysis

Image datasets were transferred to a MATLAB (The Mathworks, Natick, USA) workstation and processed offline. For all datasets, T_2_^*^ was estimated based on a linear equation set obtained from the logarithm of Equation 1, where S_(0)_ was estimated through S_(TEmin)_. The T_2_^*^ and S_0_ estimation was used as initialization values to fit the data points to a mono exponential decay (Equation 1) based on the MATLAB region trust algorithm.
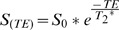
(1)


For T_2_^*^ assessment of the phantom data, a ROI covering the entire central axial view of the phantom was used. Average and standard deviation of T_2_^*^ were determined.

For examination of the *in vivo* data, an affine registration of the **MB CINE** datasets was incorporated into the post-processing procedure to compensate for misalignments due to the use of multiple breath-hold periods. The affine registration is landmark based. It shifts and shears the datasets derived from multiple breath-held acquisitions. Landmarks were set manually. Mid-ventricular short axis T_2_^*^ maps were segmented according to the standardized myocardial segmentation and nomenclature for tomographic imaging of the heart [45]. For each segment of the mid-ventricular slice (segment 7–12 according to [45]), T_2_^*^ values were calculated during end-diastole and end-systole for the single cardiac phase approach. For assessment of temporal changes in T_2_^*^ throughout the cardiac cycle ROIs encompassing segment 7 to 12 were defined and analysed for all cardiac phases derived from MB CINE. For this purpose, the position and shape of the ROI was carefully adjusted throughout the cardiac cycle to account for myocardial contraction and relaxation. Also, this approach was used to include only compact myocardium into the analysis so that blood or trabecular tissue contributions can be eliminated. For careful delineation of the myocardial borders 2D CINE FLASH (flip angle  = 32°, acquisition data matrix  = 256×224, FOV  =  (288×252) mm^2^, in-plane resolution  =  (1.1×1.1) mm^2^, slice thickness = 4 mm, TE = 2.8 ms, TR = 4.2 ms) was used. Mean values and standard deviation of T_2_^*^ were calculated for all ROIs. Statistical analysis was performed to test for data distribution and group differences using R project for statistical computing (OpenSource: www.r-project.org). A p-value below 0.05 was considered as statistically significant.

## Results

### Phantom Studies

**Figure 2:**
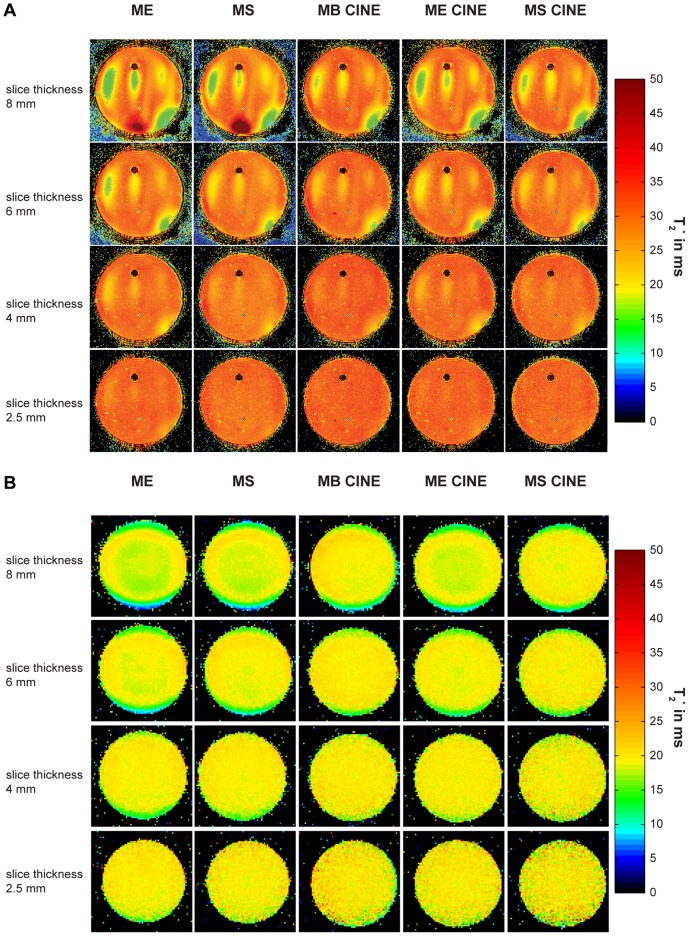
Survey of T_2_^*^ maps derived from phantom studies. T_2_^*^ maps obtained for all imaging strategies using a long T_2_^*^ (**A**) and a medium T_2_^*^phantom (**B**). Slice thicknesses ranging from 8 mm to 2.5 mm (top to bottom) were applied. T_2_^*^ analysis revealed similar results for all T_2_^*^ mapping strategies. For a slice thickness of 8 mm T_2_^*^ varied substantially across both phantoms. The uniformity in T_2_^*^ was improved for a slice thickness of 6 mm and even further enhanced for a slice thickness of 4 mm or 2.5 mm.

T_2_^*^ maps derived with all imaging strategies for the phantom experiments are surveyed in Figure 2 and Table 2 and show that all imaging strategies provide similar T_2_^*^ maps. For the long T_2_^*^ phantom, T_2_^*^ varied from 13.6 ms to 37.4 ms across the entire central axial slice when using a slice thickness of 8 mm. The non-uniformity in T_2_^*^ was reduced for 6 mm slices with T_2_^*^ ranging from 19.7 ms to 33.9 ms across the entire central axial slice. T_2_^*^ non-uniformity across the central axial slice was further reduced upon further reducing the slice thickness to 4 mm and 2.5 mm, which showed T_2_^*^ of (29.1±1.5) ms and (29.9±1.7) ms, respectively. T_2_^*^ values derived from a ROI (d = 2 cm) placed in the iso-center of the central axial slice of the phantom varied between 26.1 ms (**ME**) and 28.3 ms (**MB CINE**) for a slice thickness of 8 mm. In comparison, T_2_^*^ values ranging from 28.7 ms (**MS**) to 30.7 (**MB CINE**) were observed for the same small ROI when using a slice thickness of 2.5 mm. T_2_^*^ values derived from CINE imaging remained constant (std <1 ms) throughout the cycle given by the gating paradigm.

**Table 2:** Survey of T_2_^*^ derived from phantom studies for single cardiac phase and for CINE T_2_^*^ mapping techniques.

	Mapping technique				
Slice thickness	ME mean±std	MS mean±std	MB CINE mean±std	ME CINE mean±std	MS CINE mean±std
			(temporal std)	(temporal std)	(temporal std)
[mm]	[ms]	[ms]	[ms]	[ms]	[ms]
**8**	26.1±3.7	26.7±3.0	28.3±2.8	26.9±3.2	27.2±2.6
			(0.4)	(0.4)	(0.3)
	18.3±0.8	18.6±0.8	19.5±0.9	18.6±0.7	19.3±0.7
			(0.3)	(0.1)	(0.3)
**6**	27.5±2.5	27.2±1.8	28.5±1.9	27.9±2.3	27.6±1.7
			(0.4)	(0.5)	(0.4)
	19.0±0.6	19.2±0.7	19.7±0.7	19.2±0.6	19.5±0.9
			(0.4)	(0.2)	(0.4)
**4**	28.6±1.6	27.7±1.3	30.2±1.5	29.0±1.5	28.7±1.2
			(0.5)	(0.5)	(0.5)
	19.4±0.7	19.3±0.6	19.8±1.0	19.5±0.8	19.7±1.3
			(0.7)	(0.4)	(0.7)
**2.5**	30.0±1.3	28.7±1.1	30.7±1.3	30.2±1.3	29.8±1.3
			(0.7)	(0.6)	(0.8)
	19.6±0.9	19.5±2.2	20.1±1.7	19.9±1.3	20.1±2.1
			(1.2)	(0.6)	(1.2)

Mean T_2_^*^ and standard deviation of T_2_^*^ derived from **ME, MS, ME CINE, MS CINE** and **MB CINE** acquisitions using a slice thickness ranging from 8 mm to 2.5 mm. For all slice thicknesses the top rows show T_2_^*^ for the long T_2_^*^ phantom while the bottom rows show T_2_^*^ for the medium T_2_^*^ phantom. For the long T_2_^*^ phantom T_2_^*^ was observed for a ROI (diameter 2 cm) placed in the iso-center of an axial slice of the phantom. For the mediumT_2_^*^ phantom T_2_^*^ was observed for a ROI (diameter 6 cm). Please note, for CINE protocols temporal T_2_^*^ variation is given in parentheses as standard deviation of mean T_2_^*^ over the CINE cycle.

For the medium T_2_^*^ phantom, mean T_2_^*^ varied between 18.3 ms and 19.3 ms for a ROI covering the entire central axial slice (slice thickness = 8 mm). For a slice thickness of 6 mm, mean T_2_^*^ values were ranging from 19.0 ms to 19.9 ms. T_2_^*^ mapping using a slice thickness of 4 mm yielded mean T_2_^*^ values ranging from 19.3 ms to 19.8 ms. For a 2.5 mm slice thickness the range of mean T_2_^*^ values encompassed 19.5 ms to 20.1 ms. For this slice thickness the standard deviation of T_2_^*^ across the central slice of the phantom was approximately 2 ms for **MS, MB and MS CINE** due to SNR constraints. T_2_^*^ values derived from CINE imaging remained constant (std <1 ms) throughout the cycle given by the cardiac gating paradigm.

In all phantom experiments, the acquisition time of the **MS** approach was doubled versus the **ME** approach, as summarized in Table 1. In **MS,** only 5 views per segment were recorded while **ME** used 10 views per segment. This approach has been deliberately chosen already at this stage to ensure that the acquisition windows do not exceed the cardiac rest period in the *in vivo* studies. For **ME** **CINE,** only two views per segment were used to accomplish an acquisition window of 38 ms which increased the total scan duration to 121 s in the phantom studies. This scan duration was doubled for **MS CINE** since only one view per segment could be used for this approach.

### Volunteer Studies

**Figure 3:**
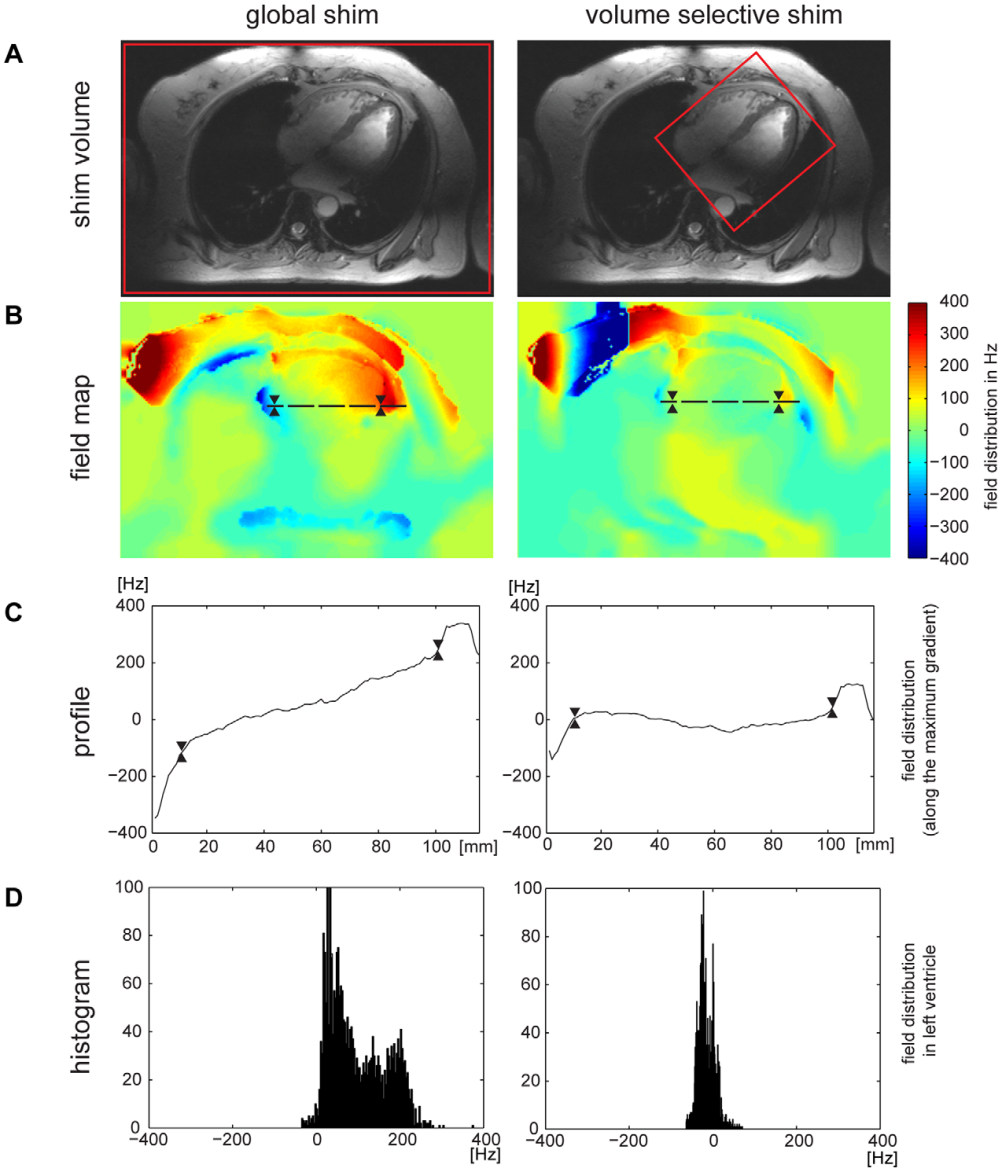
B_0_ distribution for global and volume selective B_0_ shimming of a four chamber view of the heart. **A**) Four chamber view of the heart illustrating the positioning of the volume (marked in red) used for global (left) and volume selective (right) shimming. **B**) B_0_ field variation derived from global and volume selective shimming. For this subject the global shim provided a peak-to-peak field variation of about 400 Hz across the entire heart. After volume selective shimming peak-to-peak B_0_ variation across the heart was reduced to approximately 80 Hz. The direction of the maximal B_0_ gradient is illustrated by the dashed black line in **B**) and the corresponding profile of B_0_ field distribution is plotted in **C**). To guide the eye the epicardial borders are marked in **B**) and **C**) by two triangles. The histogram of the field distribution over the left ventricle is shown in **D**). The full width at half maximum is approximately 200 Hz for the globally shimmed B_0_ field map and was reduced to about 80 Hz after volume selective shimming.

**Figure 4:**
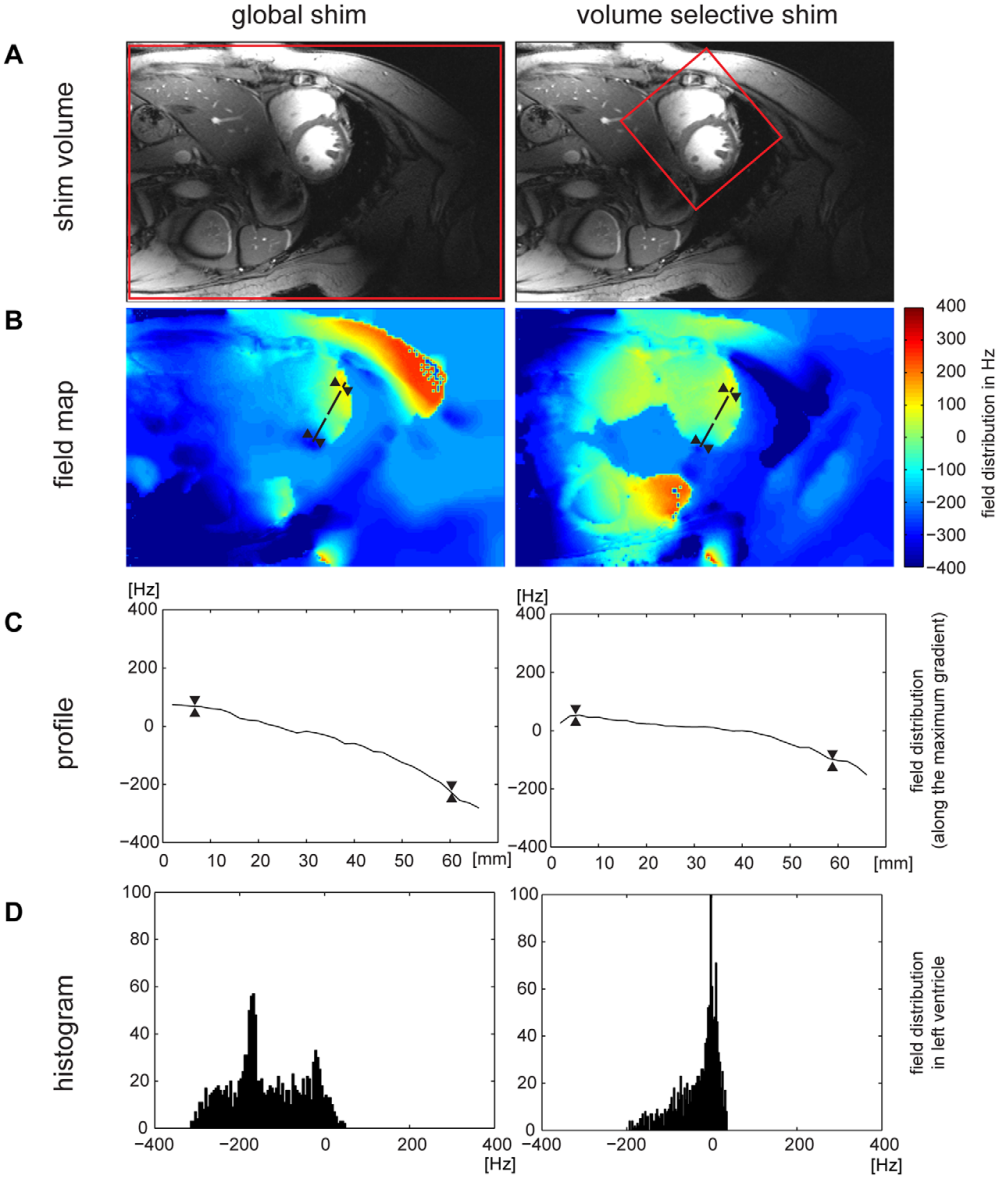
B_0_ distribution for global and volume selective shimming of a mid-ventricular short axis view of the heart. **A**) Mid-ventricular short axis view of the heart illustrating the positioning of the volume (marked in red) used for volume selective shimming. **B**) B_0_ field maps. **C**) B_0_ profile along the direction of the strongest B_0_ gradient which is highlighted by the dashed black line in **B**). To guide the eye the epicardial borders are marked in **B**) and **C**) by two triangles. **D**) Frequency histogram across the left ventricle. After volume selective shimming a strong susceptibility gradient at the inferior region of the heart could be reduced. The full width at half maximum is approximately 300 Hz for the globally shimmed field map and was reduced to about 80 Hz after volume selective shimming.

For the *in vivo* studies, **MS** and **MB CINE** were applied using a slice thickness of 4 mm to balance the competing constraints between SNR and B_0_ background gradients. A reduction in slice thickness helps to reduce intra-voxel dephasing due to B_0_ gradients along the slice direction. This slice thickness is afforded by the SNR advantage inherent to 7.0 T and is smaller than that commonly used for T_2_^*^ mapping at 1.5 T and 3.0 T. Localized shimming was performed to reduce static magnetic field inhomogeneities to make sure that the susceptibility weighting is not dominated by macroscopic B_0_ field inhomogeneities but rather by microscopic B_0_ susceptibility gradients. Figure 3 and Figure 4 depict B_0_ maps together with B_0_ profiles across the heart and frequency histograms of the heart obtained prior to and after volume selective shimming for a four chamber (Figure 3) and short axis view (Figure 4). The B_0_ field maps following global shimming showed a mean peak-to-peak field difference of approximately 400 Hz across the heart for a four chamber view (Figure 3B) and approximately 300 Hz for a mid-ventricular short axis view (Figure 4B). After volume selective shimming, a mean peak-to-peak B_0_ difference of approximately 80 Hz was found across the entire heart for a four chamber view (Figure 3B) and a mid-ventricular short axis view (Figure 4B) of the heart. For the left ventricle a B_0_ peak-to-peak difference of approximately 65 Hz was observed after volume selective shimming. For both short axis view and 4 chamber view, a maximum in-plane field gradient of approximately 20 Hz/mm (through-plane approximately 80 Hz/voxel for a 4 mm slice thickness) was observed at the epicardial fat/lung interface of the inferior and inferolateral segment as indicated by the B_0_ maps and frequency profiles shown in Figure 3B,C and Figure 4B,C. This local B_0_ gradient translates into a phase loss of approximately 80% at the maximum echo time of TE = 10 ms. However, the through-plane field gradient at the epicardium/lung interface is much more pronounced versus the through-plane field gradient obtained for the left and right ventricle, as demonstrated in Figure 3B/C. For myocardial anterior, anterolateral and inferoseptal segments a mean in-plane B_0_ gradient of approximately 3 Hz/mm was obtained which translates into an through-plane B_0_ dispersion of approximately 12 Hz/voxel for a 4 mm slice thickness. This B_0_ gradient implies that macroscopic intravoxel dephasing effects are of minor effect for the TE range used. Inspite of B_1_^+^ shaping using EMF simulations the signal intensity change across the myocardium of a mid-ventricular short axis slice was found to be approximately 40%.

**Figure 5:**
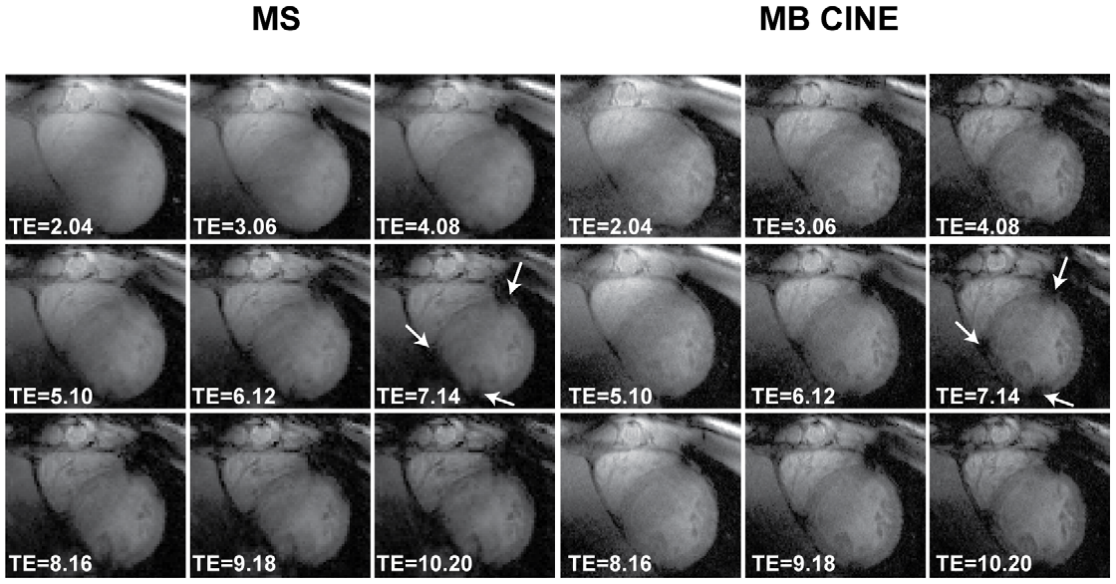
Short axis views derived from single cardiac phase and dynamic CINE T_2_^*^ weighted imaging of the heart. Echo times ranging from 2.04 ms to 10.20 ms were used for **MS** and **MB CINE** acquisitions. A low nominal flip angle of 20° was used to preserve myocardial signal. Image quality observed for **MS** and **MB CINE** acquisitions is comparable**.** No severe susceptibility artifacts were detected in the septum and in the lateral wall for TEs ranging between 2.04 ms to 10.20 ms. For anterior and inferior myocardial areas encompassing major cardiac veins susceptibility weighting related signal void was observed for TE >7 ms as highlighted by white arrows.

We next employed **MS** and **MB CINE** to determine the limits of susceptibility artifacts. Figure 5 shows end-diastolic short axis views derived from **MS** and **MB CINE** acquisitions using echo times ranging from 2.04 ms to 10.20 ms. With the flip angle of 20°, RF power deposition was well in the SAR limits given by the IEC guidelines [46], which were confirmed by rigorous electromagnetic field simulations [36], [37]. The low flip angle was deliberately chosen to preserve myocardial signal by reducing T_1_-saturation effects. Although this approach results in a low contrast between the blood pool and the surrounding myocardium (Figure 5), it is beneficial for myocardial T_2_^*^ quantification. Unlike previous reports at 1.5 T and 3.0 T double inversion recovery prepared blood suppression was not used since this approach does not meet the requirements of CINE T_2_^*^mapping. It should be noted that no differences in mean T_2_^*^ values were found for black blood versus white blood acquisitions at 1.5 T [47]. No severe susceptibility artifacts were detected in the septum (segment 8–9) and in the lateral wall (segment 11–12) for TEs ranging between 2.04 ms to 10.20 ms. For anterior (segment 7) and inferior (segment 10) myocardial areas, which encompass major cardiac veins, a signal void related to the susceptibility weighting was observed for TE >7 ms (Figure 5).

**Figure 6:**
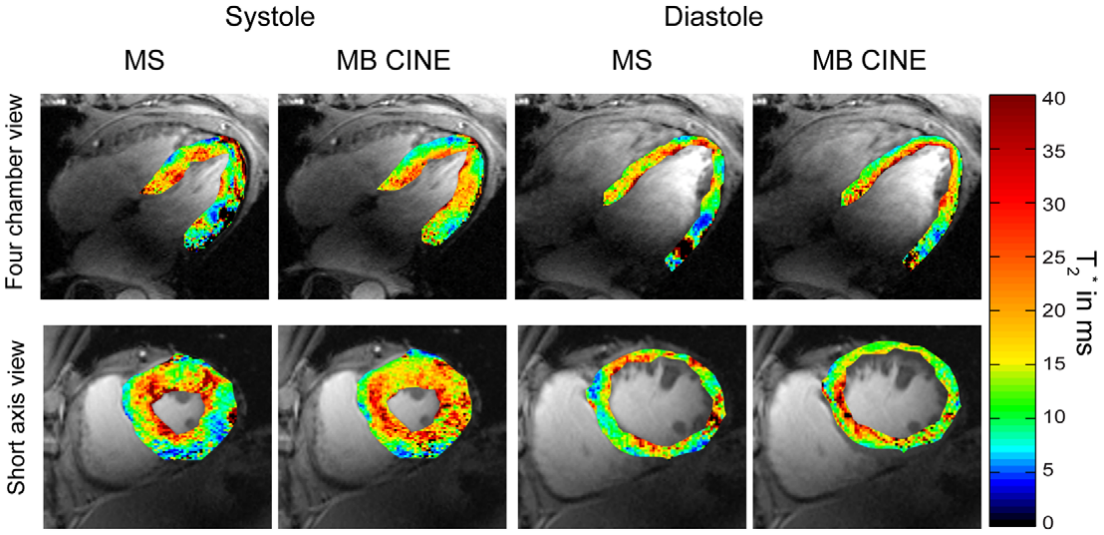
T_2_^*^ maps derived from single cardiac phase and dynamic CINE mapping of a four chamber and short axis view of the heart at end-diastole and end-systole. Four chamber (top) and short axis view T_2_^*^ colour maps obtained from **MS** and **MB CINE** superimposed to anatomical 2D CINE FLASH gray scale images. For **MB CINE** a systolic and diastolic phase was chosen to match the cardiac phase with the end-systolic and end-diastolic phase derived from **MS**. T_2_^*^ maps deduced from **MS** and **MB CINE** showed no significant differences between both methods in the segmental analysis of T_2_^*^ values. When comparing systolic and diastolic T_2_^*^ maps significant differences were found with p = 0.002 for MS and p = 0.01 for **MB CINE**.

We next explored myocardial T_2_^*^ using single cardiac phase (**MS**) and dynamic CINE (**MB CINE**) acquisitions. Figure 6 shows end-systolic and end-diastolic short axis and four chamber view T_2_* maps. Regions of T_2_^*^ reduction were observed in areas adjacent to the large cardiac vein and the posterior vein. A paired t-test showed no significant differences in T_2_* values observed for **MS** and **MB CINE** strategies. (p = 0.8 for diastole, p = 0.7 for systole). The differences between the **MS** the **MB CINE** imaging strategy including all mid-ventricular segments were found to be T_2_*_diff_ = (0.2±3.7) ms at end-diastole and T_2_*_diff_ = (−0.2±3.4) ms at end-systole. **MS** and **MB CINE** showed a mean T_2_^*^ of approximately 14 ms for mid-ventricular myocardium. Regional T_2_^*^ variation was observed for mid-ventricular myocardium. The longest T_2_^*^ values were found for segment 8 (**MS:** T_2_^*^ = 17.2 ms, **MB CINE:** T_2_^*^ = 17.3 ms), segment 9 (**MS:** T_2_^*^ = 16.5, **MB CINE:** T_2_^*^ = 16.3) and segment 7 (**MS:** T_2_^*^  = 14.0 ms, **MB CINE:** T_2_^*^ = 16.8 ms). For segment 10 and segment 11 lower T_2_^*^ values were observed (segment 10: **MS:** T_2_^*^ = 10.6 ms, **MB CINE:** T_2_^*^ = 12.0 ms, segment 11: **MS:** T_2_^*^ = 11.4 ms, **MB CINE:** T_2_^*^ = 11.4 ms). A synopsis of T_2_^*^ values averaged over all subjects for **MS** and **MB CINE** acquisitions at end-diastole is given in Table 3.

**Table 3:** Summary of mean and standard deviation of T_2_^*^ (in ms) at end-diastole and at end-systole.

	cardiac segment					
	7	8	9	10	11	12
**MB CINE end-systole**	13.7±2.9	17.4±2.5	14.8±1.8	10.5±4.2	8.3±2.4	10.9±1.7
**MB CINE end-diastole**	16.8±2.2	17.3±1.4	16.3±2.2	12.0±3.6	11.4±2.8	12.5±1.9
**MS end-systole**	12.4±2.1	17.2±2.7	15.7±2.9	7.6±2.1	10.2±2.0	13.6±1.9
**MS end-diastole**	14.0±1.8	17.2±2.6	16.5±2.0	10.6±4.4	11.4±2.5	15.7±2.0

Mean T_2_^*^ (in ms) averaged over all subjects for each cardiac segment of a mid-ventricular short axis derived from single cardiac phase **MS** and from **MB CINE** acquisitions at end-diastole and at end-systole. T_2_^*^ values obtained for both approaches show a fair agreement. The statistical analysis showed no significant difference between T_2_^*^ derived from **MS** and T_2_^*^ deduced from **MB CINE** acquisitions.

**Figure 7:**
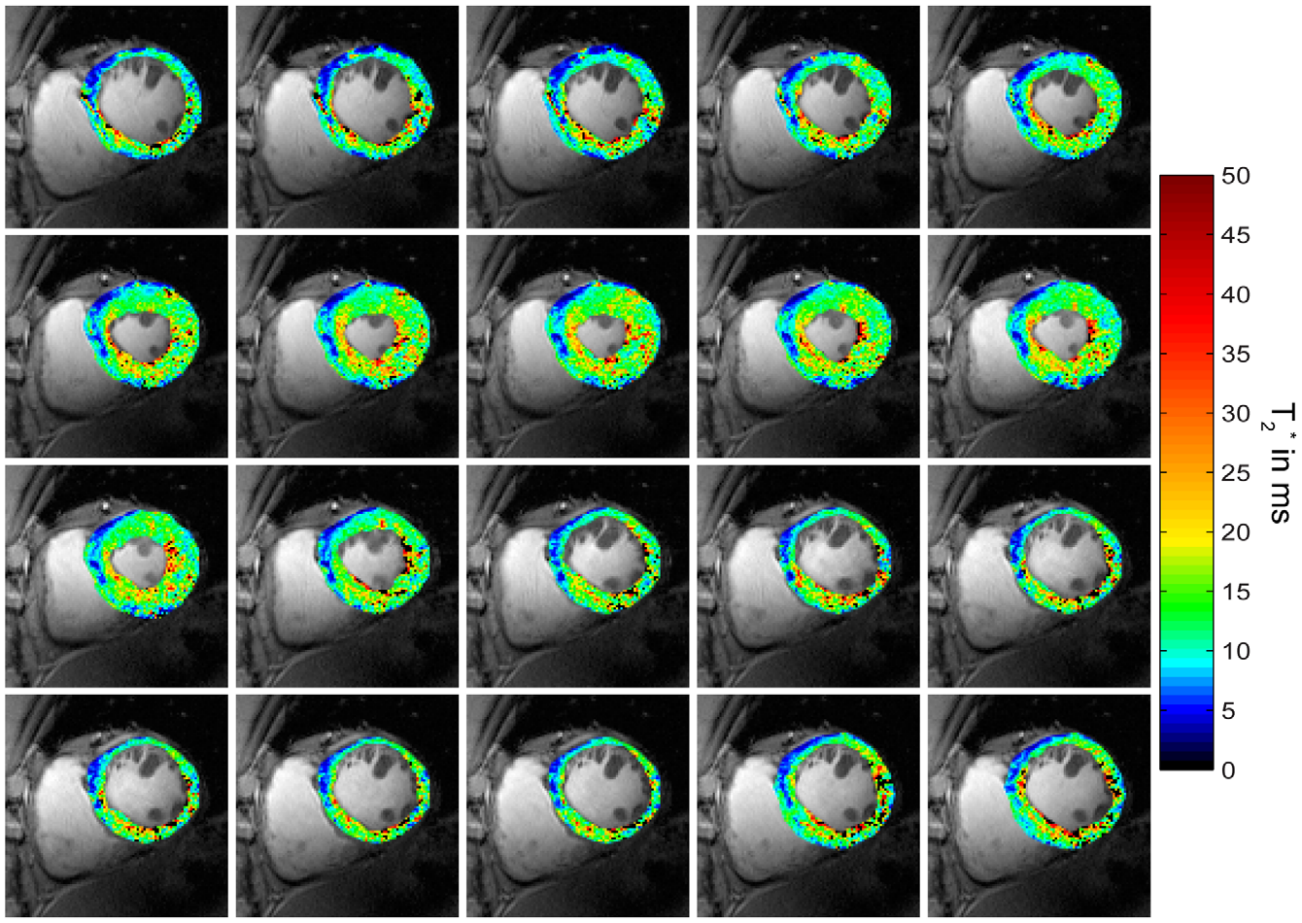
CINE T_2_^*^ maps over the cardiac cycle. Short axis view T_2_^*^ colour maps derived from **MB CINE** acquisitions across the cardiac cycle overlaid to conventional 2D CINE FLASH images. T_2_^*^ values are increasing from diastole to systole, especially for endocardial layers. Macroscopic susceptibility induced T_2_^*^ reduction effects were present at the epicardium at inferior regions.

**Figure 8:**
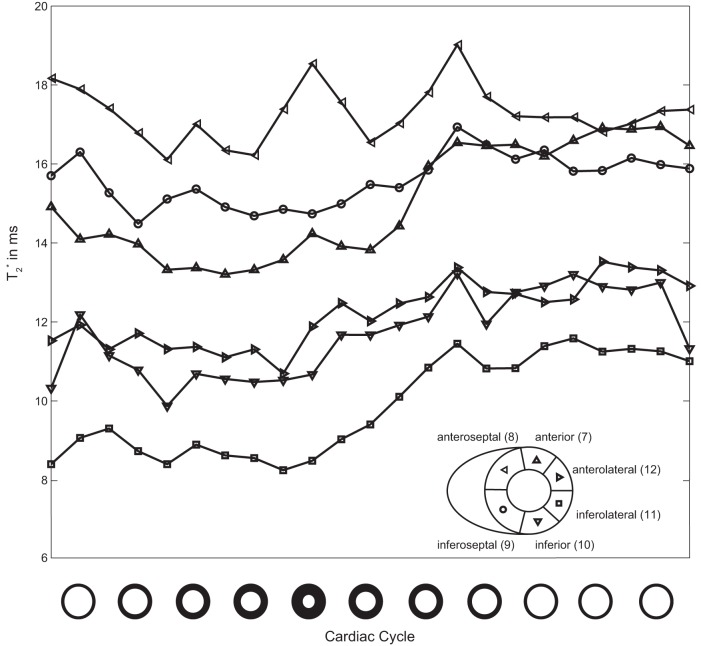
Analysis of T_2_^*^ across the cardiac cycle. Synopsis of the evolution of mean T_2_^*^ averaged over all subjects for standard mid-ventricular segments of the heart. T_2_^*^ derived from each cardiac segment are plotted versus the cardiac cycle. T_2_^*^ changes over the cardiac cycle. Averaging T_2_^*^ over all mid-ventricular myocardial segments revealed that T_2_^*^ increases approximately 27% between systole and diastole. Myocardial T_2_^*^ was derived from **MB CINE** acquisitions. Prospective triggering was used which resulted in a gap at end-diastole of approximately 100 ms depending on the heart rate. For this reason the cardiac cycle is normalized for all subjects without including this gap.

A closer examination revealed a significant difference between myocardial T_2_^*^ obtained at end-diastole and those derived from end-systolic acquisitions. Indeed, an increase in T_2_^*^ can be clearly observed, particularly at the septum, during systolic phases (Figure 6, Figure 7). Figure 7 shows exemplary CINE T_2_^*^ maps for all phases of the cardiac cycle and illustrates the changes of T_2_^*^ over the cardiac cycle. A paired t-test comparing end-diastolic and end-systolic phase T_2_^*^ values for all segments revealed p = 0.002 for single cardiac phase **MS** acquisitions and p = 0.01 for **MB CINE** acquisitions. When averaging the T_2_^*^ time course from all subjects an increase in T_2_^*^ of 27%±6% in all cardiac segments could be observed over the cardiac cycle (Figure 8). The shortest T_2_^*^ values were noted for a cardiac phase placed in systole. For all cardiac segments, the longest T_2_^*^ values were found after the onset of diastole. The largest T_2_^*^ increase over the cardiac cycle was found for segment 7 (ΔT_2_^*^  = +3.7 ms) and segment 10 (ΔT_2_^*^  = +3.4 ms).

**Figure 9:**
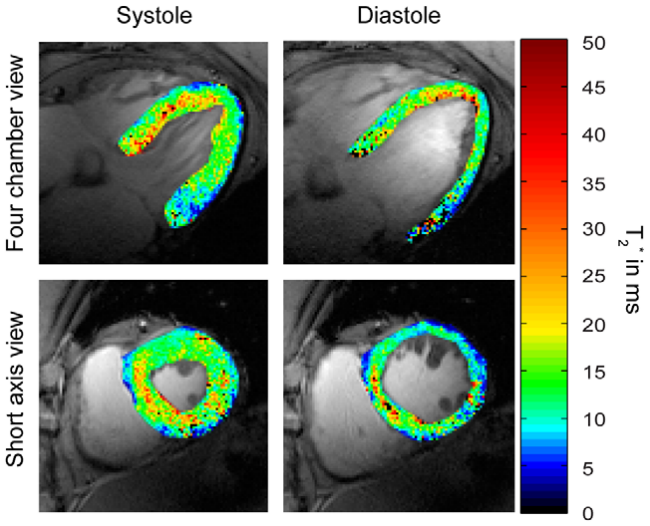
High spatial resolution four chamber and short axis view T_2_^*^ maps derived from T2* weighted CINE imaging. For **MB CINE** slice thickness was reduced to 2.5 mm while maintaining the in-plane spatial resolution of (1.1×1.1) mm^2^. Compared to the results obtained with **MB CINE** using a slice thickness of 4 mm, changes in T_2_^*^ from epicardial to endocardial septal myocardial layers are more pronounced, in particular during systole.

To demonstrate the baseline SNR advantage of 7.0 T and to further reduce intravoxel dephasing along the slice direction, the slice thickness was reduced to 2.5 mm while maintaining the in-plane spatial resolution of (1.1×1.1) mm^2^ in the **MB** CINE protocol. Figure 9 shows short axis and four chamber view T_2_^*^ maps, which demonstrate the high spatial resolution. This approach further manifests the observed changes in T_2_^*^ during systole and diastole and also indicates the high sensitivity of susceptibility mapping by visualizing differences in T_2_^*^ between endocardial and epicardial layers of the myocardium.

## Discussion

This work shows the feasibility of high spatially and temporally resolved myocardial T_2_^*^ mapping at 7.0 T. For this purpose T_2_^*^ weighted, gradient echo based imaging techniques using single cardiac phase (**MS**) and CINE (**MB CINE**) acquisition regimes were benchmarked against T_2_^*^ mapping techniques commonly used in current clinical practice at 1.5 T and 3.0 T. These two imaging techniques were first examined in detail in phantom experiments.

It might be considered as a remaining limitation that our results might be affected by residual macroscopic B_0_ gradients. However, our B_0_ mapping results suggest that a reasonable B_0_ uniformity across the heart and the left ventricle can be achieved at 7.0 T which is embodied by a mean through-plane gradient of 3 Hz/mm across the left ventricle. In this regard it should be also noted that our measurements of the B_0_ field distribution after volume selective shimming of our uniform phantom provided a through slice peak-to-peak B_0_ variation of 80 Hz along a distance of 4 cm which translates into 2 Hz/mm. This B_0_ field gradient is similar to what has been observed for the left and right ventricle which showed a mean of 3 Hz/mm.

The frequency shift across the heart reported here compares well with previous 3.0 T studies which reported a peak-to-peak off-resonance variation of (262±58) Hz over the left ventricle and the right ventricle (basal short axis view) for a global shim [48]. This B_0_ inhomogeneity was improved to (176±30) Hz and (121±31) Hz with the use of localized linear and second-order shimming [48]. The use of an enhanced locally optimized shim algorithm, which is tailored to the geometry of the heart, afforded a reduction of the peak-to-peak frequency variation over the heart from 235 Hz to 86 Hz at 3.0 T [49]. Another pioneering study showed a peak-to-peak off-resonance of (71±14) Hz for short axis views acquired at 1.5 T [40] using global shimming.

Previous reports on brain imaging/spectroscopy suggest that third-order and even higher order shims help to further enhance B_0_ uniformity across the target anatomy [50], [51]. For this purpose extra shim drum inserts are retrofitted to the scanner. Notwithstanding its utility the current implementation limits the available space inside of the MR scanner bore which would be prohibitive for cardiac or body MRI at 7.0 T. Other attempts to integrate third order shim coils into high performance 7.0 T whole body gradient coil designs were found to show pronounced gradient non-linearity for spherical volumes with a diameter larger than (20–25) cm; a behavior which does not meet the requirements of cardiac or body MR. Obviously, another approach to further reduce the residual impact of through-plane gradients and intra-voxel dephasing B_0_ gradients is the use of even thinner slices and the reduction in voxel size. To meet this goal we pushed the envelope by using a slice thickness as thin as 2.5 mm together with an in-plane resolution of (1.1×1.1) mm^2^. This slice thickness and in-plane resolution is afforded by the SNR advantage inherent to 7.0 T. The corresponding voxel size is by a factor of five smaller than commonly used for T_2_^*^ mapping at 1.5 T and 3.0 T. However, it should be noted that the move to even thinner slices and smaller voxel sizes – ideally one might opt to use an infinitesimal small voxel – would disturb the balance dictated by the competing constraints of SNR and background gradients effects.

This study sheds further light to the current literature since it demonstrates the applicability of **MS** and **MB CINE** for T_2_^*^ mapping of normal myocardium at 7.0 T. While we recognize a limitation due to the limited number of healthy subjects studied, we believe this feasibility study to be an essential precursor to a larger 7.0 T study involving healthy and patient cohorts. Such a study would aid to establish the lower limits for normal myocardial T_2_^*^ values versus the clinically established normal values for T_2_^*^ of healthy myocardium at 1.5 T and 3.0 T. To this end, T_2_^*^ mapping at 7.0 T may be useful to extend the capabilities and the dynamic range of the sensitivity of the established approach used for quantification of myocardial iron content. With this in mind, we anticipate to extend our efforts towards clinical studies at 7.0 T including thalassemia major patients, whose T_2_^*^ relaxation times will be benchmarked against the normal values of healthy subjects.

Our results show that T_2_^*^ obtained for human myocardial muscle tissue at 7.0 T ranges from 9 ms to 18 ms. This is in line with T_2_^*^  = (15.8±0.2) ms recently observed for hind limb skeletal muscle in rats at 7 Tesla [52] Admittedly, the absolute spatial resolution demonstrated for T_2_^*^ mapping of the human heart at 7.0 T is still by an order of magnitude below that previously reported for *ex vivo* MR microscopy based T_2_^*^mapping of the isolated rat heart [7], which demonstrated that T_2_^*^mapping provides an insight into the complex architecture of the heart musculature. However, the effective anatomical spatial resolution – voxel size per anatomy – is getting close to what has been demonstrated for animal models. This improvement might be beneficial to gain a better insight into the myocardial microstructure *in vivo* with the ultimate goal to visualize myocardial fibers or to examine helical angulation of myocardial fibers using T_2_^*^ mapping, since the susceptibility effects depend on the tilt angle between blood filled capillaries and the external magnetic field [53]. Myocardial fibre tracking using T_2_^*^ mapping holds the promise to be less sensitive to bulk motion than diffusion-weighted MR of the myocardium [54], [55]. Our results also suggest that the increased susceptibility contrast available at 7.0 T could be exploited to quantitatively study iron accumulations in organs other than the heart with high sensitivity and temporal and spatial resolution superior to what can be achieved at 1.5 T and 3.0 T.

For normal myocardium a T_2_^*^ value of approximately 37 ms was found at 1.5 T [23]. At 3.0 T a T_2_^*^ of approximately 27 ms was observed for normal myocardium [56]. These measurements are usually limited to the septum, which shows the lowest spatial variation in T_2_^*^ [57]. It is elusive to study temporal changes in T_2_^*^ at 1.5 and 3.0 T due to scan time constraints which are prohibitive for CINE T_2_^*^ mapping. Of course, single cardiac phase T2* mapping can be applied to diastole and systole as reported previously [58]. This 1.5 T study with thalassemia patients demonstrated mean T_2_^*^ values of (26.4±14.2) ms for early systole and (25.2±13.1) ms for late diastole, which were found to be not significantly different (P = 0.27). However, the limited T_2_^*^ sensitivity together with the temporal resolution used in the study presents a challenge for tracking temporal changes in T_2_^*^. Please also note, that these data exhibit a rather large standard deviation of approximately ±13.0 ms, which presents another challenge for assessment of temporal T_2_^*^ changes.

A careful literature research revealed that no 1.5 T and 3.0 T T_2_^*^ mapping study has been reported yet which uses a high spatial resolution accomplished here. We would also like to point out that our study is the first study which affords CINE T_2_^*^ mapping due to inter echo time shortening at 7.0 T. To this end it is interesting to note that the myocardial BOLD effect has been investigated using SSFP imaging, which is sensitive to changes in the relaxation times T_2_ and T_1_. In this regard it has been shown recently that the signal intensity derived from SSFP imaging of the myocardium varies across the cardiac cycle [59]. This study showed a systole-to-diastole T_2_ ratio of approximately 1.1 for normal myocardium.

The ability to probe for changes in tissue oxygenation using T_2_^*^ sensitized imaging/mapping offers the potential to address some of the spatial and temporal resolution constraints of conventional first pass perfusion imaging and holds the promise to obviate the need for exogenous contrast agents. Since microscopic susceptibility increases with field strength, thus making the BOLD effect due to (patho)physiology of interest more pronounced, T_2_^*^ mapping at 7.0 T might be beneficial to address some of the BOLD sensitivity constraints reported for the assessment of regional myocardial oxygenation changes in the presence of coronary artery stenosis [60] or for the characterization of vasodilator-induced changes of myocardial oxygenation at 1.5 T and at 3.0 T [17].

## Conclusion

Our results underscore the challenges of myocardial T_2_^*^ mapping at 7.0 T due to the propensity to macroscopic susceptibility artefacts and T_2_^*^ shortening, but demonstrate that these issues can be offset by using tailored shimming techniques together with dedicated acquisition schemes.
